# Supplementary material for: Correction of z‐motion artefacts to allow population imaging of synaptic activity in behaving mice
Source: J Physiol. 2020 Mar 3;598(10):1809–27. doi: 10.1113/JP278957 (PMC7318612; doi:10.1113/JP278957)
Supplement: Supplementary file 1 — Statistical Summary Document [file TJP-598-1809-s001.docx]

**Manuscript Title:** Correction of z-motion artefacts to allow population imaging of synaptic activity in behaving mice

**Authors:** Thomas Ryan, Antonio Hinojosa, Rozan Vroman, Christoforos Papasavvas, Leon Lagnado.

**Animal model used, if applicable:** Mouse primary visual cortex – C57 (WT) and transgenic (VIP:CRE) on CBA/Ca background, with virally delivered SyGCaMP6f expression under CRE.

**Underlying hypothesis:** *In-vivo* functional imaging in awake animals is susceptible to artefacts caused by axial motion of the imaging plane that can be corrected by procedures described in this study.

**Definitions of ‘n’:**

Question 1: n = values of R0 of individual Moffat function fits to individual synapses. Are they significantly affected by ongoing activity?

Question 2: n = values of FWHM of a Moffat function fit to an individual synapse. Are they significantly affected by ongoing activity?

**Statistical summary table:**

| Experimental question number* | Conclusion | Experimental variable | Mean value | SD | N (mice) | n (fields of view) | n (synapses) | P** | Units | Data comparisons | Statistical test | Figure/ table in which data are presented |
| --- | --- | --- | --- | --- | --- | --- | --- | --- | --- | --- | --- | --- |
| Difference in FWHM values in RSF fits with and without activity? | Similar values of FWHM | With activity | 7.060 | 1.81375 | 2 | 3 | 34 | 0.063 | μm | With activity vs. without activity | Wilcoxon Ranked Sum test | 6 |
|  |  | Without activity | 7.325 | 1.256 | 2 | 3 | 34 | 0.063 | μm |  |  | 6 |

*You may use multiple lines for the same question to indicate multiple comparisons

** Authors may wish to make the text bold where p is considered significant against a stated confidence limit.
